# (Ethano­lato-κ*O*)[*N*′-(3-meth­oxy-2-oxidobenzyl­idene-κ*O* ^2^)benzo­hydrazidato-κ^2^ *N*′,*O*]oxidovanadium(V)

**DOI:** 10.1107/S1600536812032229

**Published:** 2012-07-21

**Authors:** Xiao-Hua Chen, Qiong-Jie Wu, Li-Juan Chen, Ming-Xing Yang

**Affiliations:** a College of Materials Science and Engineering, Fujian Normal University, Fuzhou, Fujian 350007, People’s Republic of China; b College of Life Science, Fujian Agriculture and Forestry University, Fuzhou, Fujian 350002, People’s Republic of China; c College of Chemistry and Chemical Engineering, Fujian Normal University, Fuzhou, Fujian 350007, People’s Republic of China

## Abstract

In the title complex, [V(C_15_H_12_N_2_O_4_)(C_2_H_5_O)O], the V^V^ ion is coordinated by an oxide O atom, an ethano­late anion and two O atoms and one N atom from the tridentate benzo­hydrazidate dianion in a distorted square-pyramidal geometry; the V atom is displaced by 0.4748 (8) Å from the basal plane towards the axial oxide O atom. An intra­molecular O—H⋯N hydrogen bond occurs in the benzohydrazidate ligand. Weak inter­molecular C—H⋯O hydrogen bonding is present in the crystal.

## Related literature

For general background to the coordination chemistry and biochemisty of vanadium, see: Deng *et al.* (2007 ▶); Monfared *et al.* (2011 ▶); Sutradhar *et al.* (2006 ▶). For related structures, see: Chen *et al.* (2004 ▶); Liu *et al.* (2006 ▶); Ghosh *et al.* (2007 ▶); Seena *et al.* (2008 ▶). For the synthesis, see: Gao *et al.* (1998 ▶); Huang *et al.* (2010 ▶).
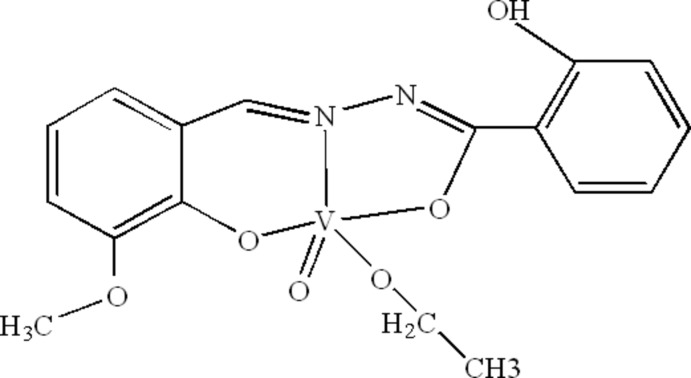


## Experimental

### 

#### Crystal data

[V(C_15_H_12_N_2_O_4_)(C_2_H_5_O)O]
*M* *_r_* = 396.27
Monoclinic, 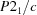

*a* = 15.808 (5) Å
*b* = 6.606 (2) Å
*c* = 16.693 (8) Å
β = 94.107 (16)°
*V* = 1738.6 (12) Å^3^
*Z* = 4
Mo *K*α radiation
μ = 0.61 mm^−1^
*T* = 293 K
0.37 × 0.25 × 0.13 mm

#### Data collection

Rigaku R-AXIS RAPID diffractometer
Absorption correction: multi-scan (*TEXRAY*; Molecular Structure Corporation, 1999 ▶) *T*_min_ = 0.834, *T*_max_ = 0.924
15371 measured reflections
3968 independent reflections
3243 reflections with *I* > 2σ(*I*)
*R*_int_ = 0.041

#### Refinement

*R*[*F* ^2^ > 2σ(*F* ^2^)] = 0.038
*wR*(*F* ^2^) = 0.105
*S* = 1.08
3968 reflections
237 parameters
H-atom parameters constrained
Δρ_max_ = 0.34 e Å^−3^
Δρ_min_ = −0.22 e Å^−3^

### 

Data collection: *TEXRAY* (Molecular Structure Corporation, 1999 ▶); cell refinement: *TEXRAY*; data reduction: *TEXSAN* (Molecular Structure Corporation, 1999 ▶); program(s) used to solve structure: *SHELXS97* (Sheldrick, 2008 ▶); program(s) used to refine structure: *SHELXL97* (Sheldrick, 2008 ▶); molecular graphics: *ORTEX* (McArdle, 1995 ▶); software used to prepare material for publication: *SHELXL97*.

## Supplementary Material

Crystal structure: contains datablock(s) I, global. DOI: 10.1107/S1600536812032229/xu5587sup1.cif

Structure factors: contains datablock(s) I. DOI: 10.1107/S1600536812032229/xu5587Isup2.hkl

Additional supplementary materials:  crystallographic information; 3D view; checkCIF report

## Figures and Tables

**Table 1:** Selected bond lengths (Å)

V1—N1	2.1029 (15)
V1—O1	1.8325 (14)
V1—O3	1.9453 (14)
V1—O5	1.5762 (15)
V1—O6	1.7423 (13)

**Table 2:** Hydrogen-bond geometry (Å, °)

*D*—H⋯*A*	*D*—H	H⋯*A*	*D*⋯*A*	*D*—H⋯*A*
O4—H4*B*⋯N2	0.82	1.86	2.581 (2)	147
C8—H8*A*⋯O4^i^	0.93	2.31	3.236 (2)	177

Symmetry code: (i) 
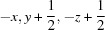
.

**Fig. 1.**
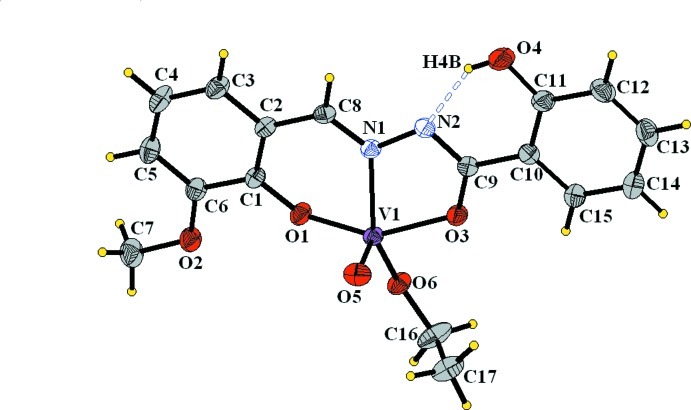
The molecular structure of the title compound, showing 30% probability displacement ellipsoids for non-H atoms. A dashed line indicates the intramoleculat hydrogen bonding.

## supplementary crystallographic information

### Comment

In the recent years, the coordination chemistry and biochemisty of vanadium has received considerable attention (Deng *et al.*, 2007; Monfared *et al.*, 2011; Sutradhar *et al.*, 2006). Generally, a tridentate hydrazone ligand is coordinated to the vanadium through O and N atoms, similar to those of the biological system. So, it is important to intensively study the relation ship of the syntheses and structural properties of vanadium hydrazone complexes.

In the title complex, [VO(C_15_H_12_N_2_O_4_)(C_2_H_5_O)], the V^V^ ion exists in a distorted square-pyramidal coordination geometry. Three donor atoms (O1, O3 and N1) of the hydrozone ligand and O6 atom from the ethanol group define the coordination basal plane, with a maximum mean plane deviation of 0.030 (1) Å. The V atom is displaced towards the axial oxo O atom by 0.4748 (8) Å from the basal plane. Bond distances (Table 1) and bond angles around V1 atom are compared with those in reported oxovanadium complexes (Chen *et al.*, 2004; Seena *et al.*, 2008; Liu *et al.*,2006; Ghosh *et al.*, 2007). In the crystal structure there are the intramolecular O—H···N hydrogen bonding and intermolecular C—H···O hydrogen bonding (Table 2).

### Experimental

VO(acac)_2_ (acac = acetylacetonate) was synthesized according to the reported method of Gao *et al.* (1998). The synthesis of the hydrazone ligand has already been reported in the literature (Huang *et al.*, 2010).

The title compound was prepared by reacting H_2_L (0.1 mmol) with VO(acac)_2_ (0.1 mmol) in ethanol solvent with stirring. The solution was filtered and allowed to stand at room temperature for one week, and dark-red crystals of complex (I) were obtained.

### Refinement

All H atoms were placed in idealized positions and treated as riding with O—H = 0.82 Å, C—H = 0.93–0.97 Å; *U*_iso_(H) = 1.2*U*_eq_(C) or 1.5*U*_eq_(C, O).

### Crystal data

**Table:**

[V(C_15_H_12_N_2_O_4_)(C_2_H_5_O)O]	*F*(000) = 816
*M**_r_* = 396.27	*D*_x_ = 1.514 Mg m^−^^3^
Monoclinic, *P*2_1_/*c*	Mo *K*α radiation, λ = 0.71073 Å
Hall symbol: -P 2ybc	Cell parameters from 3243 reflections
*a* = 15.808 (5) Å	θ = 3.3–27.5°
*b* = 6.606 (2) Å	µ = 0.61 mm^−^^1^
*c* = 16.693 (8) Å	*T* = 293 K
β = 94.107 (16)°	Prism, dark-red
*V* = 1738.6 (12) Å^3^	0.37 × 0.25 × 0.13 mm
*Z* = 4	

### Data collection

**Table:**

Rigaku R-AXIS RAPID diffractometer	3968 independent reflections
Radiation source: fine-focus sealed tube	3243 reflections with *I* > 2σ(*I*)
Graphite monochromator	*R*_int_ = 0.041
ω scans	θ_max_ = 27.5°, θ_min_ = 3.3°
Absorption correction: multi-scan (*TEXRAY*; Molecular Structure Corporation, 1999)	*h* = −19→20
*T*_min_ = 0.834, *T*_max_ = 0.924	*k* = −8→7
15371 measured reflections	*l* = −21→21

### Refinement

**Table:**

Refinement on *F*^2^	Primary atom site location: structure-invariant direct methods
Least-squares matrix: full	Secondary atom site location: difference Fourier map
*R*[*F*^2^ > 2σ(*F*^2^)] = 0.038	Hydrogen site location: inferred from neighbouring sites
*wR*(*F*^2^) = 0.105	H-atom parameters constrained
*S* = 1.08	*w* = 1/[σ^2^(*F*_o_^2^) + (0.0564*P*)^2^ + 0.3657*P*] where *P* = (*F*_o_^2^ + 2*F*_c_^2^)/3
3968 reflections	(Δ/σ)_max_ = 0.001
237 parameters	Δρ_max_ = 0.34 e Å^−^^3^
0 restraints	Δρ_min_ = −0.22 e Å^−^^3^

### Special details

**Table:**

Geometry. All e.s.d.'s (except the e.s.d. in the dihedral angle between two l.s. planes) are estimated using the full covariance matrix. The cell e.s.d.'s are taken into account individually in the estimation of e.s.d.'s in distances, angles and torsion angles; correlations between e.s.d.'s in cell parameters are only used when they are defined by crystal symmetry. An approximate (isotropic) treatment of cell e.s.d.'s is used for estimating e.s.d.'s involving l.s. planes.
Refinement. Refinement of *F*^2^ against ALL reflections. The weighted *R*-factor *wR* and goodness of fit *S* are based on *F*^2^, conventional *R*-factors *R* are based on *F*, with *F* set to zero for negative *F*^2^. The threshold expression of *F*^2^ > σ(*F*^2^) is used only for calculating *R*-factors(gt) *etc*. and is not relevant to the choice of reflections for refinement. *R*-factors based on *F*^2^ are statistically about twice as large as those based on *F*, and *R*- factors based on ALL data will be even larger.

### Fractional atomic coordinates and isotropic or equivalent isotropic displacement parameters (Å^2^)

**Table:**

	*x*	*y*	*z*	*U*_iso_*/*U*_eq_	
V1	0.327165 (17)	0.03858 (5)	0.379657 (17)	0.03227 (11)	
O1	0.30812 (8)	0.2237 (2)	0.45811 (8)	0.0442 (3)	
O2	0.35894 (10)	0.5229 (2)	0.55498 (9)	0.0496 (4)	
O3	0.28941 (8)	−0.2038 (2)	0.32138 (8)	0.0407 (3)	
O4	0.04552 (9)	−0.2451 (3)	0.20545 (12)	0.0782 (6)	
H4B	0.0647	−0.1583	0.2368	0.117*	
O5	0.36775 (9)	0.1609 (2)	0.31098 (9)	0.0516 (4)	
O6	0.40591 (8)	−0.1056 (2)	0.43088 (8)	0.0435 (3)	
N1	0.19698 (9)	0.0812 (2)	0.34927 (8)	0.0328 (3)	
N2	0.15908 (9)	−0.0609 (2)	0.29735 (9)	0.0372 (4)	
C1	0.25579 (11)	0.3816 (3)	0.46463 (10)	0.0357 (4)	
C2	0.17609 (11)	0.3877 (3)	0.42268 (10)	0.0358 (4)	
C3	0.12022 (13)	0.5497 (3)	0.43503 (12)	0.0472 (5)	
H3A	0.0668	0.5530	0.4077	0.057*	
C4	0.14474 (15)	0.7014 (4)	0.48709 (13)	0.0546 (6)	
H4A	0.1078	0.8079	0.4952	0.065*	
C5	0.22441 (14)	0.6982 (3)	0.52816 (12)	0.0488 (5)	
H5A	0.2402	0.8029	0.5633	0.059*	
C6	0.28039 (13)	0.5414 (3)	0.51739 (11)	0.0394 (4)	
C7	0.38639 (16)	0.6801 (4)	0.60921 (14)	0.0617 (6)	
H7A	0.4451	0.6602	0.6263	0.092*	
H7B	0.3531	0.6772	0.6551	0.092*	
H7C	0.3795	0.8087	0.5828	0.092*	
C8	0.14914 (11)	0.2298 (3)	0.36837 (11)	0.0366 (4)	
H8A	0.0938	0.2341	0.3453	0.044*	
C9	0.21338 (11)	−0.2052 (3)	0.28567 (10)	0.0342 (4)	
C10	0.18783 (11)	−0.3739 (3)	0.23241 (10)	0.0353 (4)	
C11	0.10557 (12)	−0.3869 (4)	0.19530 (13)	0.0471 (5)	
C12	0.08370 (15)	−0.5527 (4)	0.14672 (14)	0.0570 (6)	
H12A	0.0287	−0.5640	0.1234	0.068*	
C13	0.14231 (15)	−0.6997 (4)	0.13284 (13)	0.0541 (5)	
H13A	0.1269	−0.8090	0.0998	0.065*	
C14	0.22383 (15)	−0.6862 (3)	0.16759 (13)	0.0509 (5)	
H14A	0.2636	−0.7851	0.1575	0.061*	
C15	0.24613 (13)	−0.5261 (3)	0.21720 (11)	0.0409 (4)	
H15A	0.3010	−0.5186	0.2411	0.049*	
C16	0.47854 (16)	−0.2211 (5)	0.41625 (15)	0.0726 (8)	
H16A	0.4651	−0.3632	0.4225	0.087*	
H16B	0.5233	−0.1876	0.4569	0.087*	
C17	0.51099 (16)	−0.1928 (5)	0.33759 (16)	0.0734 (8)	
H17A	0.5644	−0.2613	0.3359	0.110*	
H17B	0.5186	−0.0510	0.3279	0.110*	
H17C	0.4713	−0.2476	0.2970	0.110*	

### Atomic displacement parameters (Å^2^)

**Table:**

	*U*^11^	*U*^22^	*U*^33^	*U*^12^	*U*^13^	*U*^23^
V1	0.02768 (17)	0.03613 (19)	0.03235 (17)	0.00497 (12)	−0.00236 (11)	−0.00102 (12)
O1	0.0396 (7)	0.0454 (8)	0.0458 (7)	0.0120 (6)	−0.0079 (5)	−0.0127 (6)
O2	0.0487 (8)	0.0503 (8)	0.0481 (8)	0.0044 (7)	−0.0073 (6)	−0.0160 (7)
O3	0.0343 (7)	0.0413 (7)	0.0453 (7)	0.0066 (6)	−0.0066 (5)	−0.0083 (6)
O4	0.0364 (8)	0.0960 (14)	0.0991 (14)	0.0110 (9)	−0.0166 (8)	−0.0531 (11)
O5	0.0450 (8)	0.0587 (10)	0.0510 (8)	0.0002 (7)	0.0032 (6)	0.0117 (7)
O6	0.0391 (7)	0.0507 (8)	0.0391 (7)	0.0148 (6)	−0.0069 (5)	−0.0039 (6)
N1	0.0296 (7)	0.0363 (8)	0.0319 (7)	0.0023 (6)	−0.0013 (6)	−0.0012 (6)
N2	0.0315 (8)	0.0398 (9)	0.0393 (8)	0.0004 (7)	−0.0037 (6)	−0.0058 (7)
C1	0.0397 (9)	0.0352 (9)	0.0327 (9)	0.0047 (8)	0.0053 (7)	−0.0010 (7)
C2	0.0365 (9)	0.0382 (10)	0.0329 (9)	0.0065 (8)	0.0047 (7)	0.0011 (7)
C3	0.0441 (11)	0.0514 (13)	0.0454 (11)	0.0150 (9)	−0.0011 (9)	−0.0037 (9)
C4	0.0640 (14)	0.0497 (13)	0.0499 (12)	0.0238 (11)	0.0034 (10)	−0.0062 (10)
C5	0.0661 (13)	0.0419 (11)	0.0384 (10)	0.0080 (10)	0.0030 (9)	−0.0084 (9)
C6	0.0464 (11)	0.0398 (10)	0.0319 (9)	0.0032 (8)	0.0024 (7)	−0.0022 (8)
C7	0.0697 (15)	0.0599 (15)	0.0529 (13)	0.0004 (12)	−0.0131 (11)	−0.0198 (11)
C8	0.0298 (8)	0.0419 (10)	0.0376 (9)	0.0043 (8)	−0.0002 (7)	0.0021 (8)
C9	0.0347 (9)	0.0370 (10)	0.0306 (8)	0.0002 (8)	0.0003 (7)	0.0020 (7)
C10	0.0381 (9)	0.0386 (10)	0.0294 (8)	−0.0028 (8)	0.0037 (7)	0.0005 (7)
C11	0.0383 (10)	0.0558 (12)	0.0471 (11)	−0.0018 (9)	0.0030 (8)	−0.0124 (10)
C12	0.0467 (12)	0.0691 (16)	0.0548 (13)	−0.0144 (11)	0.0012 (10)	−0.0193 (11)
C13	0.0692 (15)	0.0495 (13)	0.0442 (11)	−0.0149 (11)	0.0076 (10)	−0.0117 (9)
C14	0.0667 (14)	0.0398 (11)	0.0464 (11)	0.0052 (10)	0.0061 (10)	−0.0048 (9)
C15	0.0467 (11)	0.0382 (10)	0.0376 (10)	0.0027 (8)	0.0008 (8)	0.0014 (8)
C16	0.0608 (15)	0.094 (2)	0.0636 (15)	0.0445 (15)	0.0118 (12)	0.0132 (14)
C17	0.0587 (15)	0.087 (2)	0.0769 (18)	0.0242 (14)	0.0189 (13)	0.0034 (15)

### Geometric parameters (Å, º)

**Table:**

V1—N1	2.1029 (15)	C5—C6	1.382 (3)
V1—O1	1.8325 (14)	C5—H5A	0.9300
V1—O3	1.9453 (14)	C7—H7A	0.9600
V1—O5	1.5762 (15)	C7—H7B	0.9600
V1—O6	1.7423 (13)	C7—H7C	0.9600
O1—C1	1.340 (2)	C8—H8A	0.9300
O2—C6	1.356 (2)	C9—C10	1.464 (3)
O2—C7	1.424 (3)	C10—C15	1.399 (3)
O3—C9	1.303 (2)	C10—C11	1.402 (3)
O4—C11	1.353 (3)	C11—C12	1.391 (3)
O4—H4B	0.8200	C12—C13	1.373 (3)
O6—C16	1.414 (2)	C12—H12A	0.9300
N1—C8	1.293 (2)	C13—C14	1.378 (3)
N1—N2	1.385 (2)	C13—H13A	0.9300
N2—C9	1.307 (2)	C14—C15	1.374 (3)
C1—C2	1.397 (3)	C14—H14A	0.9300
C1—C6	1.411 (3)	C15—H15A	0.9300
C2—C3	1.412 (3)	C16—C17	1.456 (3)
C2—C8	1.427 (3)	C16—H16A	0.9700
C3—C4	1.364 (3)	C16—H16B	0.9700
C3—H3A	0.9300	C17—H17A	0.9600
C4—C5	1.390 (3)	C17—H17B	0.9600
C4—H4A	0.9300	C17—H17C	0.9600
			
O5—V1—O6	108.88 (8)	O2—C7—H7C	109.5
O5—V1—O1	105.89 (9)	H7A—C7—H7C	109.5
O6—V1—O1	99.34 (7)	H7B—C7—H7C	109.5
O5—V1—O3	100.64 (8)	N1—C8—C2	123.93 (16)
O6—V1—O3	88.75 (6)	N1—C8—H8A	118.0
O1—V1—O3	147.85 (6)	C2—C8—H8A	118.0
O5—V1—N1	101.43 (7)	O3—C9—N2	121.40 (17)
O6—V1—N1	147.57 (7)	O3—C9—C10	119.24 (16)
O1—V1—N1	82.81 (6)	N2—C9—C10	119.35 (16)
O3—V1—N1	74.32 (6)	C15—C10—C11	118.54 (18)
C1—O1—V1	135.34 (12)	C15—C10—C9	120.02 (17)
C6—O2—C7	117.11 (17)	C11—C10—C9	121.44 (17)
C9—O3—V1	118.32 (12)	O4—C11—C12	118.07 (19)
C11—O4—H4B	109.5	O4—C11—C10	122.61 (19)
C16—O6—V1	140.40 (14)	C12—C11—C10	119.3 (2)
C8—N1—N2	115.75 (15)	C13—C12—C11	120.8 (2)
C8—N1—V1	128.45 (12)	C13—C12—H12A	119.6
N2—N1—V1	115.61 (11)	C11—C12—H12A	119.6
C9—N2—N1	109.18 (14)	C12—C13—C14	120.3 (2)
O1—C1—C2	121.40 (17)	C12—C13—H13A	119.8
O1—C1—C6	119.24 (17)	C14—C13—H13A	119.8
C2—C1—C6	119.32 (17)	C15—C14—C13	119.7 (2)
C1—C2—C3	119.96 (18)	C15—C14—H14A	120.1
C1—C2—C8	121.00 (17)	C13—C14—H14A	120.1
C3—C2—C8	119.03 (17)	C14—C15—C10	121.2 (2)
C4—C3—C2	119.83 (19)	C14—C15—H15A	119.4
C4—C3—H3A	120.1	C10—C15—H15A	119.4
C2—C3—H3A	120.1	O6—C16—C17	115.4 (2)
C3—C4—C5	120.60 (19)	O6—C16—H16A	108.4
C3—C4—H4A	119.7	C17—C16—H16A	108.4
C5—C4—H4A	119.7	O6—C16—H16B	108.4
C6—C5—C4	120.9 (2)	C17—C16—H16B	108.4
C6—C5—H5A	119.6	H16A—C16—H16B	107.5
C4—C5—H5A	119.6	C16—C17—H17A	109.5
O2—C6—C5	125.51 (18)	C16—C17—H17B	109.5
O2—C6—C1	115.07 (17)	H17A—C17—H17B	109.5
C5—C6—C1	119.41 (18)	C16—C17—H17C	109.5
O2—C7—H7A	109.5	H17A—C17—H17C	109.5
O2—C7—H7B	109.5	H17B—C17—H17C	109.5
H7A—C7—H7B	109.5		
			
O5—V1—O1—C1	68.39 (19)	C7—O2—C6—C5	−1.0 (3)
O6—V1—O1—C1	−178.79 (18)	C7—O2—C6—C1	−179.65 (19)
O3—V1—O1—C1	−76.1 (2)	C4—C5—C6—O2	−179.3 (2)
N1—V1—O1—C1	−31.50 (18)	C4—C5—C6—C1	−0.7 (3)
O5—V1—O3—C9	−89.56 (14)	O1—C1—C6—O2	2.8 (3)
O6—V1—O3—C9	161.46 (13)	C2—C1—C6—O2	−179.60 (17)
O1—V1—O3—C9	55.77 (18)	O1—C1—C6—C5	−175.88 (18)
N1—V1—O3—C9	9.46 (12)	C2—C1—C6—C5	1.7 (3)
O5—V1—O6—C16	−37.9 (3)	N2—N1—C8—C2	178.23 (17)
O1—V1—O6—C16	−148.3 (3)	V1—N1—C8—C2	−6.9 (3)
O3—V1—O6—C16	63.0 (3)	C1—C2—C8—N1	−5.5 (3)
N1—V1—O6—C16	120.4 (3)	C3—C2—C8—N1	175.82 (19)
O5—V1—N1—C8	−85.71 (17)	V1—O3—C9—N2	−9.5 (2)
O6—V1—N1—C8	115.25 (17)	V1—O3—C9—C10	171.59 (12)
O1—V1—N1—C8	19.13 (16)	N1—N2—C9—O3	1.2 (2)
O3—V1—N1—C8	176.30 (17)	N1—N2—C9—C10	−179.87 (15)
O5—V1—N1—N2	89.16 (14)	O3—C9—C10—C15	−3.2 (3)
O6—V1—N1—N2	−69.88 (17)	N2—C9—C10—C15	177.87 (17)
O1—V1—N1—N2	−166.00 (13)	O3—C9—C10—C11	177.21 (17)
O3—V1—N1—N2	−8.83 (11)	N2—C9—C10—C11	−1.8 (3)
C8—N1—N2—C9	−177.68 (16)	C15—C10—C11—O4	−178.9 (2)
V1—N1—N2—C9	6.78 (18)	C9—C10—C11—O4	0.7 (3)
V1—O1—C1—C2	29.7 (3)	C15—C10—C11—C12	1.9 (3)
V1—O1—C1—C6	−152.82 (15)	C9—C10—C11—C12	−178.46 (19)
O1—C1—C2—C3	175.72 (18)	O4—C11—C12—C13	178.7 (2)
C6—C1—C2—C3	−1.8 (3)	C10—C11—C12—C13	−2.0 (4)
O1—C1—C2—C8	−3.0 (3)	C11—C12—C13—C14	0.6 (4)
C6—C1—C2—C8	179.54 (17)	C12—C13—C14—C15	0.9 (3)
C1—C2—C3—C4	0.9 (3)	C13—C14—C15—C10	−0.9 (3)
C8—C2—C3—C4	179.6 (2)	C11—C10—C15—C14	−0.5 (3)
C2—C3—C4—C5	0.1 (3)	C9—C10—C15—C14	179.89 (18)
C3—C4—C5—C6	−0.2 (4)	V1—O6—C16—C17	14.1 (4)

### Hydrogen-bond geometry (Å, º)

**Table:**

*D*—H···*A*	*D*—H	H···*A*	*D*···*A*	*D*—H···*A*
O4—H4*B*···N2	0.82	1.86	2.581 (2)	147
C8—H8*A*···O4^i^	0.93	2.31	3.236 (2)	177

Symmetry code: (i) −*x*, *y*+1/2, −*z*+1/2.
